# Safety studies and viral shedding of intramuscular administration of oncolytic vaccinia virus TG6002 in healthy beagle dogs

**DOI:** 10.1186/s12917-020-02524-y

**Published:** 2020-08-25

**Authors:** Jérémy Béguin, Virginie Nourtier, Murielle Gantzer, Sandrine Cochin, Johann Foloppe, Jean-Marc Balloul, Eve Laloy, Dominique Tierny, Bernard Klonjkowski, Eric Quemeneur, Christelle Maurey, Philippe Erbs

**Affiliations:** 1 Transgene, Illkirch-Graffenstaden, France; 2 UMR Virologie, INRAE, Ecole Nationale Vétérinaire d’Alfort, ANSES, Université Paris-Est, Maisons-Alfort, France; 3 Department of Internal Medicine, Ecole Nationale Vétérinaire d’Alfort, Université Paris-Est, Maisons-Alfort, France; 4 Anatomical Pathology Unit, Biopôle, Ecole Nationale Vétérinaire d’Alfort, Université Paris-Est, 94700 Maisons-Alfort, France; 5 Oncovet Clinical Research, Loos, France

**Keywords:** Oncolytic virotherapy, Vaccinia virus, TG6002, *FCU1*, Cancer, Safety, Canine, Translational research

## Abstract

**Background:**

Cancer is a leading cause of mortality for both humans and dogs. As spontaneous canine cancers appear to be relevant models of human cancers, developing new therapeutic approaches could benefit both species. Oncolytic virotherapy is a promising therapeutic approach in cancer treatment. TG6002 is a recombinant oncolytic vaccinia virus deleted in the thymidine kinase and ribonucleotide reductase genes and armed with the suicide gene *FCU1* that encodes a protein which catalyses the conversion of the non-toxic 5-fluorocytosine into the toxic metabolite 5-fluorouracil. Previous studies have shown the ability of TG6002 to infect and replicate in canine tumor cell lines, and demonstrated its oncolytic potency in cell lines, xenograft models and canine mammary adenocarcinoma explants. Moreover, 5-fluorouracil synthesis has been confirmed in fresh canine mammary adenocarcinoma explants infected with TG6002 with 5-fluorocytosine. This study aims at assessing the safety profile and viral shedding after unique or repeated intramuscular injections of TG6002 in seven healthy Beagle dogs.

**Results:**

Repeated intramuscular administrations of TG6002 at the dose of 5 × 10^7^ PFU/kg resulted in no clinical or biological adverse effects. Residual TG6002 in blood, saliva, urine and feces of treated dogs was not detected by infectious titer assay nor by qPCR, ensuring the safety of the virus in the dogs and their environment.

**Conclusions:**

These results establish the good tolerability of TG6002 in healthy dogs with undetectable viral shedding after multiple injections. This study supports the initiation of further studies in canine cancer patients to evaluate the oncolytic potential of TG6002 and provides critical data for clinical development of TG6002 as a human cancer therapy.

## Background

Cancer is a leading cause of mortality throughout the world for both humans and dogs. The incidence of cancer ranges from 1 to 2% in the canine population and accounts for about half of the deaths in dogs older than 10 years [1, 2]. Despite progress in the diagnosis and treatment of advanced canine cancers, complete long-lasting remissions are still infrequent. Therefore, new therapeutic approaches are needed.

The use of oncolytic viruses to treat cancer is an emerging field in cancer research and therapy. Oncolytic virotherapy is a promising therapeutic option based on the ability of engineered viral vectors to selectively replicate in cancer cells leading to their lysis and to deliver genes encoding therapeutic proteins into cancer cells [3]. Many viruses have been studied in preclinical and clinical studies, including adenovirus, vaccinia virus, herpes virus, parvovirus, picornavirus and reovirus in both human and veterinary medicine [4–6]. Vaccinia virus (VACV) is a large, double stranded DNA virus, and member of the *poxviridae* family. Its ability to efficiently replicate, lyse host cells, and evade immune responses make VACV an attractive candidate for human and veterinary oncolytic virotherapy [7]. VACV has been shown to replicate and lyse tumor cells within 72 h of infection [8]. Furthermore, VACV replicates in cell cytoplasm, preventing the integration of viral DNA into host chromosomes [9]. VACV has also been shown to exhibit broad tumor tropism [8]. In human medicine, Pexa-Vec (*pexastimogene devacirepvec*, JX-594, SillaJen Biotherapeutics, Seoul, South Korea) is the most advanced VACV oncolytic product. It is derived from a VACV strain engineered to express GM-CSF and has successfully entered Phase III clinical trials [10]. An oncolytic VACV, designated as TG6002, has been developed with deletion of the thymidine kinase (*TK*) and the ribonucleotide reductase *(RR)* loci in its genome resulting in attenuated virulence and enhanced tumor-specific targeting [11]. To enhance therapeutic efficacy, the chimeric gene *FCU1* was inserted in the TG6002 genome. *FCU1* encodes a bifunctional fusion protein combining cytosine deaminase and uracil phosphoribosyltransferase activities. FCU1 converts the non-toxic prodrug 5-fluorocytosine (5-FC) into the chemotherapeutic compound 5-fluorouracil (5-FU), and further into 5-fluorouracil-monophosphate, which inhibits DNA and protein synthesis [12]. In murine xenograft mice treated by TG6002 followed by per os 5-FC administration, high levels of 5-FU were detected in tumors [11]. In this model, TG6002 in combination with 5-FC has significant antitumor efficacy against a large range of human tumors [11]. Another study has also showed relevant oncolytic features of TG6002 as an oncolytic therapy on canine cancer cell lines, mouse xenografts and canine mammary tumor explants [13]. Intratumoral injections of TG6002 in canine mammary tumor cells grafted onto mice lead to a significant decrease in tumor size [13]. Administration of 5-FC to those mice significantly improved the antitumor activity of TG6002. Finally, canine mammary tumor explants cultured with TG6002 and 5-FC, allowed the assessment of tumor necrosis, and conversion of 5-FC into 5-FU [13].

Evaluation of TG6002 in cancer-bearing dogs could be beneficial for both humans and dogs. As spontaneous canine tumors are relevant models for translational research in oncology, they can provide useful preclinical data for human medicine [14–16]. Moreover, new therapeutic strategies are needed to improve therapeutic options in veterinary medicine. Biosafety is a major issue with oncolytic viruses for both patients and the environment. VACV infection is generally associated with cutaneous pock lesions which participate in the shedding of the virus [17]. Clinical trials using attenuated oncolytic VACV in patients diagnosed with cancer have reported the development of mucocutaneous pustules after treatment [10, 18–23]. Similar lesions have been described in dogs receiving a *TK*-deleted VACV [24]. VACV is known to remain detectable in urine and feces for a long time [25–27]. Thus, before the use of TG6002 in pet dogs, evaluations of safety and viral shedding are needed. Considering the promising results obtained by the intratumoral route on xenograft models, intramuscular injections were chosen to mimic this route of administration in healthy dogs.

The present study aims at assessing the tolerability and viral shedding following intramuscular injections of escalating doses of TG6002 in healthy dogs. This study is a prerequisite for a phase 1 trial of intratumoral delivery of TG6002 in pet dogs suffering from incurable cancers.

## Results

### Single intramuscular injection of TG6002 was well tolerated in four healthy dogs

**Fig. 1:**
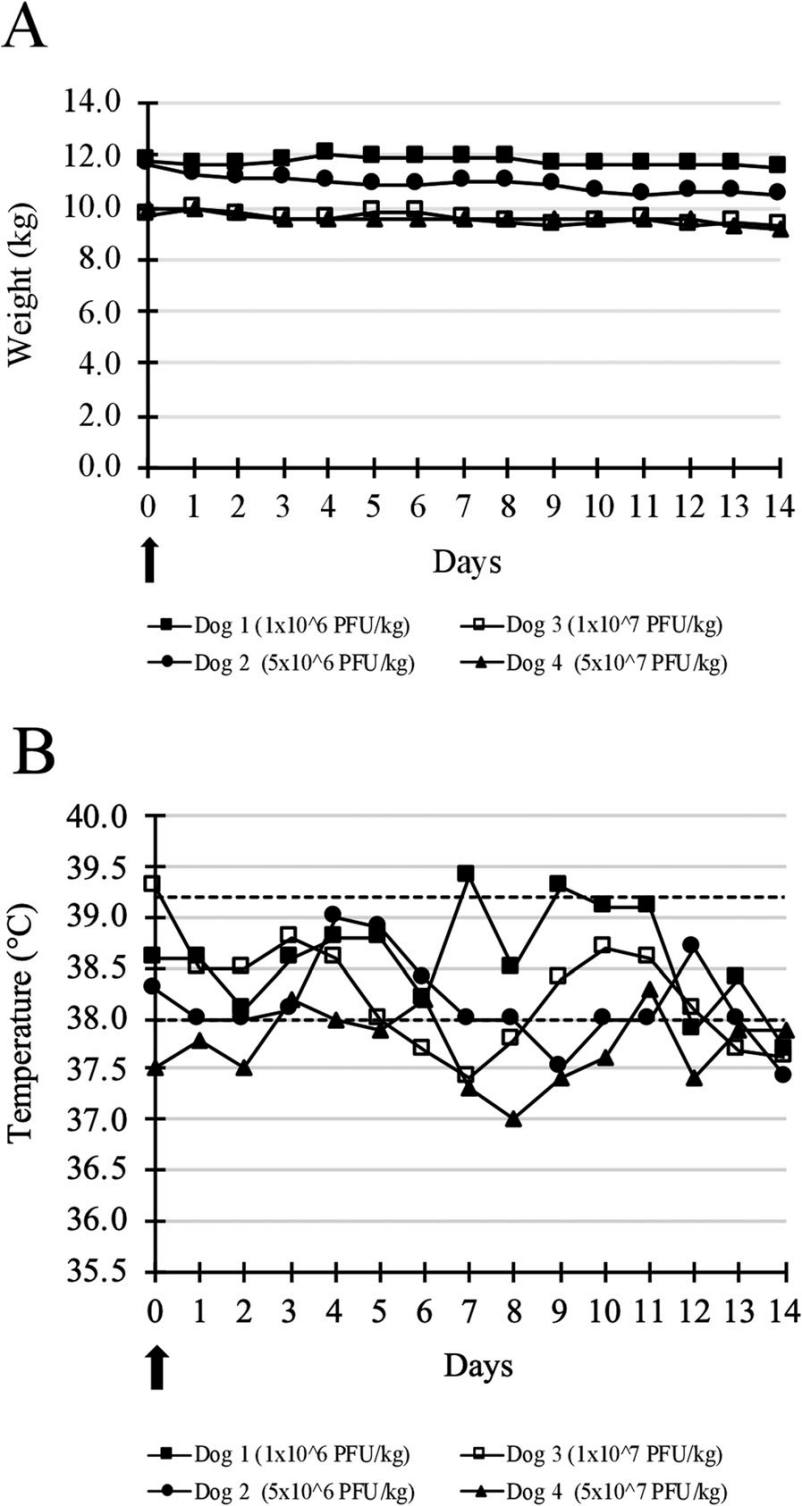
Weight (**a**) and temperature (**b**) of dogs after a single intramuscular injection of TG6002. No significant change in the weight and temperature of dogs was noticed after a single intramuscular injection of TG6002. Arrows indicate TG6002 administrations. Dotted lines represent the reference interval

**Fig. 2:**
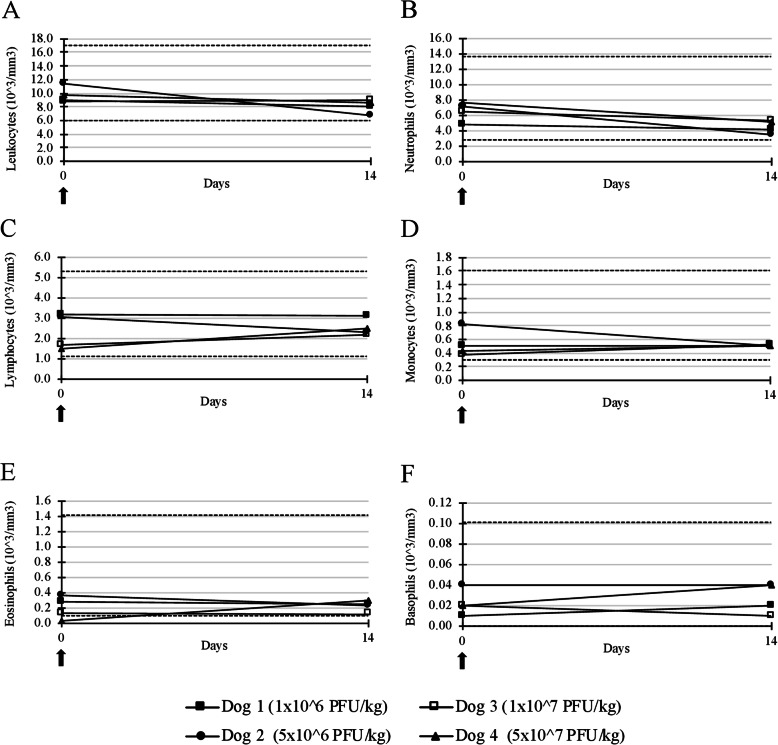
White blood cell counts of dogs after a single intramuscular injection of TG6002 TG6002 did not induce significant changes in any white blood cells after a single injection. **a**: leukocytes, **b**: neutrophils, **c**: lymphocytes, **d**: monocytes, **e**: eosinophils, **f**: basophils. Arrows indicate TG6002 administrations. Dotted lines represent the reference interval.

During the single injection phase, only a decrease in body weight scored as grade 1 was recorded for all dogs (Fig. 1a). The median percentage value of maximal weight loss was 6.05%. The decrease in body weight did not seem related to the viral titer. Only one dog (Dog 1 treated with 1 × 10^6^ PFU/kg of TG6002) had a transient increase in temperature (39.4 °C, grade 1) 7 days after TG6002 administration (Fig. 1b). Hyperthermia was not seen in the other dogs. Furthermore, no mucocutaneous or skin lesions at the injection site, nor any other clinical abnormalities were found. In addition, hematological and biochemical analyses performed on day 14 did not reveal toxicity (Fig. 2) [see Additional file 1]. For Dog 2, treated with 5 × 10^6^ PFU/kg of TG6002, total proteins were stable but in the lower part of the reference interval at days 0 and 14. For Dog 3, treated with 1 × 10^7^ PFU/kg of TG6002, a moderate decrease of total proteins was noticed between day 0 and 14 [see Additional file 1]. As maximal tolerated dose (MTD) was not reached, the highest tested dose of 5 × 10^7^ PFU/kg was selected for administration in the second phase of the study.

### Repeated intramuscular injections of 5 × 10^7^ PFU/kg of TG6002 with oral 5-FC administration were well tolerated in three healthy dogs

**Fig. 3:**
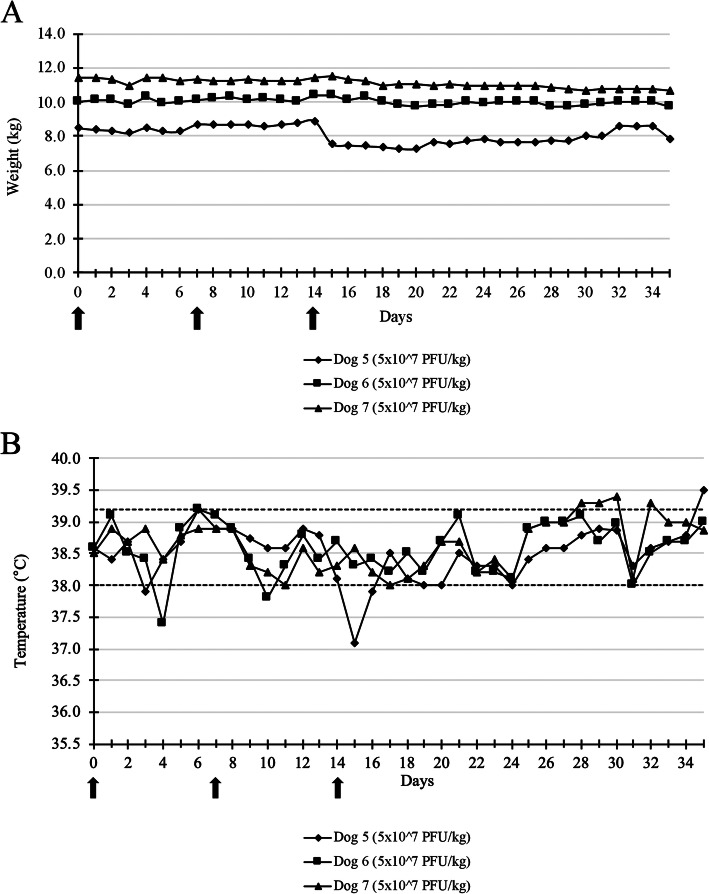
Weight (**a**) and temperature (**b**) of dogs after three intramuscular injections of TG6002. No significant change in the weight and temperature of dogs was noticed after repeated intramuscular injections of TG6002 at 5 × 10^7^ PFU/kg. Arrows indicate TG6002 administrations. Dotted lines represent the reference interval

**Fig. 4:**
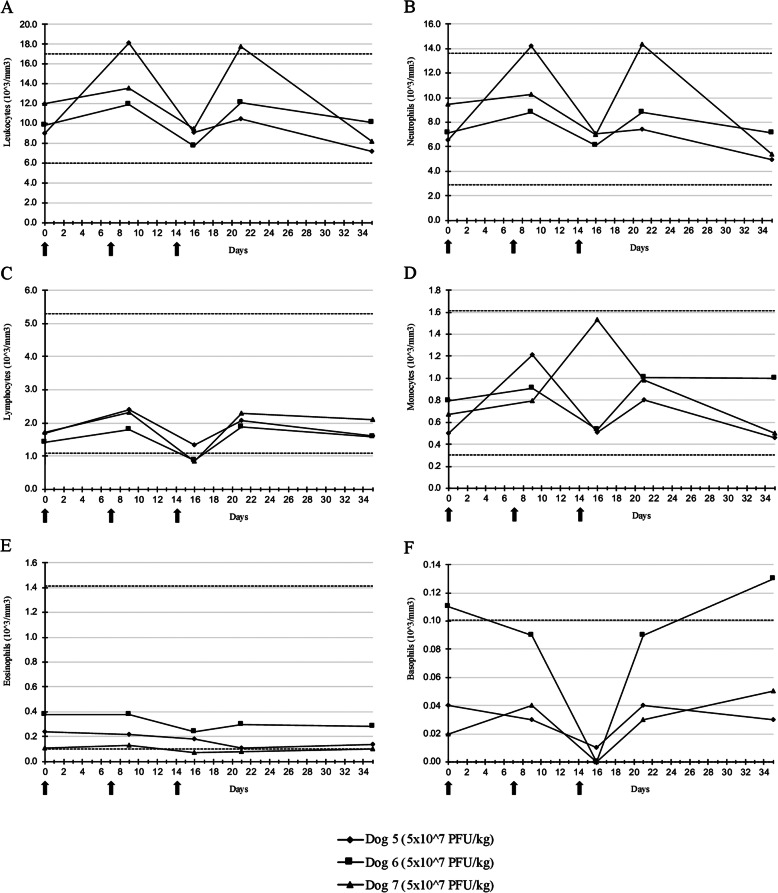
White blood cell counts of dogs after three intramuscular injections of TG6002. TG6002 did not induce significant changes in any white blood cells after repeated injections of TG6002 at 5 × 10^7^ PFU/kg. **a**: leukocytes, **b**: neutrophils, **c**: lymphocytes, **d**: monocytes, **e**: eosinophils, **f**: basophils. Arrows indicate TG6002 administrations. Dotted lines represent the reference interval

During the repeated injections phase, only a decrease in body weight scored as grade 1 was observed in two dogs (Fig. 3a). The median percentage value of maximal weight loss was 7.89%. No hyperthermia was observed (Fig. 3b). Neither mucocutaneous nor skin lesions at the injection site, nor other clinical abnormalities were found. For all dogs, an increase in the white blood cell count was noticed at days 9 and 21 (Fig. 4). On day sixteen even with values within the reference interval, a decrease in white blood cell count was noticed in all dogs. (Fig. 4). No significant abnormalities were seen in the biochemical analytes reference intervals for all dogs [see Additional file 2].

Dog 7 showed lethargy (grade 1), anorexia (grade 3), vomiting (grade 2), diarrhea (grade 2), transient melena (grade 2) and decreased body weight (grade 2) 1 day after the third injection. Analysis of serum biochemistry values revealed an increase in blood urea nitrogen (BUN) (12.70 mmol/L; reference interval: 3.30 to 10.00 mmol/L) (grade 2) with normal creatinemia (51.00 μmol/L; reference interval: 36.00 to 106.00 μmol/L) and a slight increase in alkaline phosphatase (158 UI/L; reference interval: 29 to 153 UI/L) [see Additional file 2]. No hematological abnormalities were observed (Fig. 4). Abdominal ultrasound examination was normal. Supportive care consisting of gastrointestinal protectants, a histamine type-2 receptor antagonist and supportive diet was initiated for the digestive disorders. Oral administration of kaolin and pectin (Kaopectate, Zoetis, Malakoff, France) 5 ml twice daily and sucralfate (Ulcar, Sanofi-Aventis, Gentilly, France) 1 g three times a day, with intravenous injections of ranitidine (Azantac, Laboratoire Glaxosmithkline, Marly-le-Roi, France) 1 mg/kg twice daily were dispensed. As melena was seen, an associated risk for bacterial translocation was determined, enrofloxacin (Xeden, CEVA santé Animale, Libourne, France) 5 mg/kg once daily per os was administered. Four days later, a complete resolution of clinical signs was seen and treatments were stopped at day 22. A progressive weight recovery was also observed until the thirty-fifth day. To evaluate histopathological abnormalities and viral shedding, euthanasia under general anesthesia was elected for Dog 7. Anesthesia was performed using an initial intravenous administration of 0.2 mg/kg of butorphanol (Torbugesic, Zoetis, Malakoff, France), 3 mg/kg of ketamine (Ketamine 1000, Virbac, Carros, France) and 15 μg/kg of medetomidine (Domitor, Orion Corporation, Espoo, Finland). After the dog was sedated, an intravenous injection of 180 mg/kg of sodium pentobarbital solution (Dolethal, Vetoquinol, Magny Vernois, France) was given. Postmortem examination revealed no relevant pathological changes, especially in the digestive tract, liver and kidneys. Histological analyses only revealed a slight degenerative renal tubulopathy with intra-epithelial pigment, a generalized vacuolar hepatopathy and a slight diffuse necrotic hepatitis. Immunohistochemical analyses of kidney and liver samples using VACV antibody were negative.

### Infectious VACV was not detected in blood, urine and saliva

Residual TG6002 was assessed by quantification of the infectious titer on chicken embryo fibroblasts (CEF) by plaque assay. No infectious virus was detected in blood and biological samples of treated dogs after virus administration for both parts of the study [see Additional file 3] [see Additional file 4].

### VACV DNA was not detected in blood, urine, feces and saliva

Viral DNA was not detected in blood, urine, feces, and saliva by qPCR assay for both parts of the study [see Additional file 5] [see Additional file 6]. All samples gave results below the limit of detection of the assay.

## Discussion

Oncolytic viruses are gaining ground as an alternative therapy in veterinary oncology. Several studies involving adenovirus, myxoma virus, Sendai virus, reovirus and vesicular stomatitis virus have been conducted in dogs and cats [28–36]. Promising results have been reported by intratumoral or intravenous routes on dogs and cats with cancer. No study has yet evaluated an oncolytic VACV allowing intratumoral production of a chemotherapy drug. Our study describes the dose, clinical toxicities and viral shedding after intramuscular administration of TG6002 with oral 5-FC in seven healthy immune-competent dogs. As TG6002 is engineered from VACV, evaluating both the safety and the shedding profiles of this attenuated virus remains essential. Thus, to provide more reliable information on tolerance, seven healthy dogs with a competent immune system were selected for this study. Due to ethical concerns, safety studies evaluating oncolytic viruses only involve small numbers of healthy animals [37–40]. Considering the low number of dogs in our study, it is important to remain cautious about the absence of major toxicities.

Data collected in this preclinical study on seven dogs indicate that administration of TG6002 was well tolerated in all dogs. MTD was not reached even at the highest tested dose of 5 × 10^7^ PFU/kg. Only transient weight loss for all dogs and digestive disorders for one dog out of seven were observed. One transient history of hyperthermia was noticed for one dog out of seven receiving TG6002 at the lowest dosage. During the second part of the study, a decrease in the white blood cell count was observed on day 16 for all dogs. Hematological changes were transient and values remained within the reference intervals. For all dogs (*n* = 3) receiving three injections of TG6002, increases of neutrophil, lymphocyte and monocyte counts were noticed after the second injection. An inflammatory response secondary to VACV injection was suspected. After the third injection, decreases of neutrophil, lymphocyte, monocyte, eosinophil and basophil counts were observed. Even if a stress response could not be excluded, hematological changes were suspected to be induced by viral injection. Similar variations have been described in laboratory beagles receiving intravenous administrations of an oncolytic VACV encoding CD40 ligand [37]. Leukopenia has also been reported in human trials with intratumoral oncolytic VACV. In an intratumoral dose escalation clinical trial using a Western Reserve Strain Oncolytic VACV only a grade 1or 2 leukopenia was reported in one human patient out of 16 (6.3%) treated with 1 × 10^9^ PFU [41]. In pediatric cancer patients, only a grade 3 lymphopenia was reported in one patient out of seven receiving intratumoral injection of Pexa-Vec at 1 × 10^7^ PFU/kg [42]. However, as 5-FU can induce bone marrow depression, it would have been interesting to evaluate serum concentrations of 5-FU in our study.

Other adverse events reported in laboratory beagles, receiving intravenous administration of an oncolytic VACV encoding CD40 ligand, included transient grade 1 fever (*n* = 1/2) and grade 3 seizure (*n* = 1/2) [37]. Additionally, only a grade 1 increase in alkaline phosphatase (*n* = 1/2) and a mild decrease in albumin concentration (grade 1) (*n* = 1/2) were noticed without hematological or urine abnormalities [37]. In human trials adverse events secondary to intratumoral or intravenous administration of oncolytic VACV included fever, rigors, abdominal pain, nausea, vomiting, tiredness and headache [19, 20, 42].

Infection with VACV is generally characterized by the development of cutaneous and mucocutaneous pock lesions [17]. Human cancer patients receiving attenuated oncolytic VACV have been reported to develop these lesions [10, 19, 21, 22]. Safety of a *TK*-deleted VACV (VTK-79) administered by intradermal, subcutaneous or intranasal routes has been reported in laboratory beagles [24]. Only intradermal injections of 10^7^ PFU of VTK-79 have been reported to induce the development of small nodules at the site of inoculation [24]. However, no pock lesions were observed throughout our study even if dosages were greater than 10^7^ PFU. Deletions of both *TK* and *RR* genes have been shown to improve the safety profile of VACV, due to its high attenuation in normal tissues, compared to a single deleted oncolytic vector [11]. As VACV has a tropism for cutaneous and mucocutaneous tissues, the intramuscular route of administration could also explain the lack of pock lesions.

TG6002 has a tumor-selective viral replication induced by the double *TK-RR* deletion of the TG6002 genome and does not display pathogenic effects in normal tissue [11]. No pock lesions nor any other major clinical abnormalities were observed in our study. The lack of clinical adverse events confirms the safety profile of TG6002. However, the use of healthy dogs in our study could have limited the assessment of adverse events. The dogs in this study were cancer free, but in dogs with tumors the viral load could be higher and allow viral amplification. Thus, further studies of tolerance and viral shedding must be performed in dogs with cancer.

In our study, the transient mild weight loss seen in all dogs could have been induced by virus administration. Weight loss was observed for all dogs receiving TG6002 alone (*n* = 4) or with 5-FC (*n* = 3). One dog (Dog 7) developed gastrointestinal signs which are likely related to 5-FC or TG6002. 5-FC is an antifungal agent, mainly used against strains of *Cryptococcus* and *Candida*. 5-FC penetrates fungal cells where it is deaminated by cytosine deaminase to 5-FU. It acts as an antimetabolite by competing with uracil, thereby interfering with pyrimidine metabolism and eventually RNA and protein synthesis. It is thought that 5-FU is converted into 5-fluoro-2′-deoxyuridylate which inhibits thymidylate synthesis and ultimately DNA synthesis. In humans, 5-FC is rapidly absorbed with a bioavailability of 76–89% [43]. It penetrates well into most body sites and is mostly eliminated by glomerular filtration with a 3 to 4 h half-life [44, 45]. A dose-dependent bone marrow depression (anemia, leukopenia, thrombocytopenia) and an increase in hepatic enzymes are reported [46]. Adverse events reported in dogs also include toxic epidermal necrolysis of the scrotum, nasal planum, lips and eyelids [47, 48]. These cutaneous adverse events resolve with discontinuation of 5-FC [47]. No cutaneous lesions nor hematological disorders induced by 5-FC were observed in our study. In human medicine, blood concentration should not exceed 100 mg/L within 72 h of administration of 5-FC [49]. Thus, it would have been interesting to evaluate serum concentrations of 5-FC and 5-FU in our dogs. Studies suggest that 5-FC can be converted into cytotoxic 5-FU by intestinal microorganisms leading to intestinal disorders [50, 51]. Due to the potential intestinal conversion of 5-FC into 5-FU, the dosage of 5-FU in the feces and blood would have been interesting. In a phase 1 human trial, grade 1 or 2 nausea (*n* = 6/16, 37.5%), grade 1 or 2 vomiting (*n* = 2/12; 12.5%), grade 1 or 2 diarrhea (*n* = 2/16, 12.5%) and grade 3 gastrointestinal hemorrhage (*n* = 1/16; 6.3%) were reported after intratumoral injections of oncolytic VACV [41]. For Dog 7, only an increase of BUN was noticed on biochemical analysis which was suggestive of dehydration or gastrointestinal bleeding. Post mortem examination did not reveal bowel lesions causing gastrointestinal bleeding. Even if no macroscopic intestinal lesions were observed during the necropsy, qPCR assay as well as histological and immunohistochemical analyses, would have been interesting to identify the causes of the gastrointestinal disorders.

Viral shedding detection is also important in the environmental risk assessment for this novel therapy. Viral shedding has been evaluated in dogs after intradermal or subcutaneous or intranasal administration of the *TK*-deleted VACV (VTK-79) [24]. Even if dogs with induced pock lesions were in close contact with sentinel dogs, none of these control animals developed VACV antibodies, which suggested the absence of viral shedding [24]. In a study with intravenous oncolytic VACV administrations in healthy dogs, the viral load, detected by qPCR, declined quickly in blood samples during the 4 h after infusion and viral DNA was not detected in feces, saliva and urine samples collected at 1, 2, or 4 days after virus administration [37]. In human clinical trials, viral genome has also been detected in patients’ blood after intratumoral administration of oncolytic VACV [19, 52]. In the present study, neither infectious virus nor viral genome copies were detectable in blood, urine, saliva and feces. The absence of viral shedding can be explained by the characteristics of the viral vector and the route of administration. Due to the deletion of *TK* and *RR* genes, the virus can only replicate in dividing cells [11]. Intramuscular administration of viral vector in healthy dogs was not expected to be associated with replication of the vector. As TG6002 was designed for intratumoral injection, this administration route might promote vector replication in tumors. Thus, viral burden would likely be higher in dogs with tumors that allow viral amplification. Viral replication leads to the production of FCU1 protein and the conversion of 5-FC to 5-FU. Expression of exogenous proteins without replication of the virus is considered negligible. Thus, 5-FU production was not expected after intramuscular injections in healthy dogs. In this context, tolerance and viral shedding must be evaluated further on dogs with cancer.

Previous studies evaluating oncolytic potency of TG6002 in cell lines and xenograft models have shown promising results [11, 13]. However, murine xenograft models have limitations including an impaired immune system, tumor size and the non-spontaneous origin of the tumor. Highly relevant animal models are needed to assess the efficacy of treatment before human trials. In contrast to rodents, cancers arising in dogs have several commonalities with human cancers. Dogs share the same environment as their owners, their immune system is intact, and cancer progression is spontaneous resulting in similar complexity, clonality, and immune suppression as seen in man [53]. In addition, the same cancer-associated genes and histological features have been found in both species for several cancers such as urothelial carcinoma or mammary carcinoma [54, 55]. It is now well recognized that dogs with spontaneous cancer serve as a good model for several human cancers [56]. One study has already established the oncolytic potency of TG6002 with 5-FC in canine mammary tumor explants [13]. Histological analyses of ex vivo canine mammary adenocarcinoma explants cultured with TG6002 and 5-FC, allowed assessment of tumor necrosis and conversion of 5-FC into 5-FU [13]. Considering dogs as a relevant model in oncology, and the promising in vitro results combined with the safety profile of TG6002 observed in the seven dogs in this study, intratumoral injections of 5 × 10^7^ pfu/kg of TG6002 can be considered for clinical trial in dogs with spontaneous tumors. Individual dogs will benefit from effective treatment and the results are expected to help other dogs. Furthermore, the findings are expected to help in human oncology treatments.

## Conclusion

Intramuscular injections of TG6002 at 5 × 10^7^ PFU/kg with concurrent administrations of 5-FC was well tolerated in seven healthy dogs. The evaluation of viral shedding did not reveal TG6002 excretion in the environment. These results support the future evaluation of TG6002 in pet dogs with spontaneous tumors. A veterinary study will also provide critical data for the clinical development of TG6002 as a human cancer therapy. This translational approach fits well with the “One Health – One Medicine” concept and may contribute to the development of new therapies for animal and human cancers.

## Methods

### Viral vector

TG6002 was derived from the Copenhagen strain of VACV with targeted deletions of the thymidine kinase (*J2R*) and the large subunit of ribonucleotide reductase (*I4L*) genes [11]. TG6002 expressing the fusion gene *FCU1 (ΔI4LΔJ2R/FCU1 VACV*) under the control of the p11K7.5 promoter was constructed and as previously described [11]. TG6002 was produced according to the Good Manufacturing Production on CEF, and virus stock was titrated on CEF by plaque assay.

### Laboratory dogs

Seven adult healthy male Beagle dogs (Harlan Laboratories, Gannat, France) were used. The mean weight was 10.4 ± 1.1 kg (mean ± standard deviation). All dogs were acclimatized for 7 days before the start of the experiment and had regular inspections to detect any sanitary, or behavioral events. All dogs were under the care of a licensed veterinarian. The dogs were housed individually in inox-steel bar boxes with a resin soil substrate and a softwood chips litter. The room temperature was 19 °C (+/− 2 °C) with a humidity greater than 35%, and the day/night cycle was 12:12 h. Dogs were fed twice daily with a certified commercial canine diet and given potable water ad libitum.

Dogs were not euthanized at the end of the study, but instead returned to the facility colony except in the case of grade 3 or grade 4 clinical, hematological or biochemical adverse events [57].

### Study design

This study was conducted in accordance with European legislation and French regulations on the protection of animals used for scientific purposes (Directive 2010/63/EU, 2010; Code rural, 2018; Décret no. 2013–118, 2013) and complied to the recommendations of the “Charte nationale portant sur l’éthique en expérimentation animale” established by the “Comité National de Réflexion Ethique sur l’Expérimentation Animale” (CNREEA—Ministère de l’Enseignement Supérieur, de la Recherche et de l’Innovation—Ministère de l’Agriculture et de l’Alimentation). The study protocol (n°1431_v2) was approved by the VetAgro Sup Ethical Committee (C2EA No. 18) and the Ministry of National Education, Higher Education and Research. Informed written consent to participate was required from the owners.

**Fig. 5:**
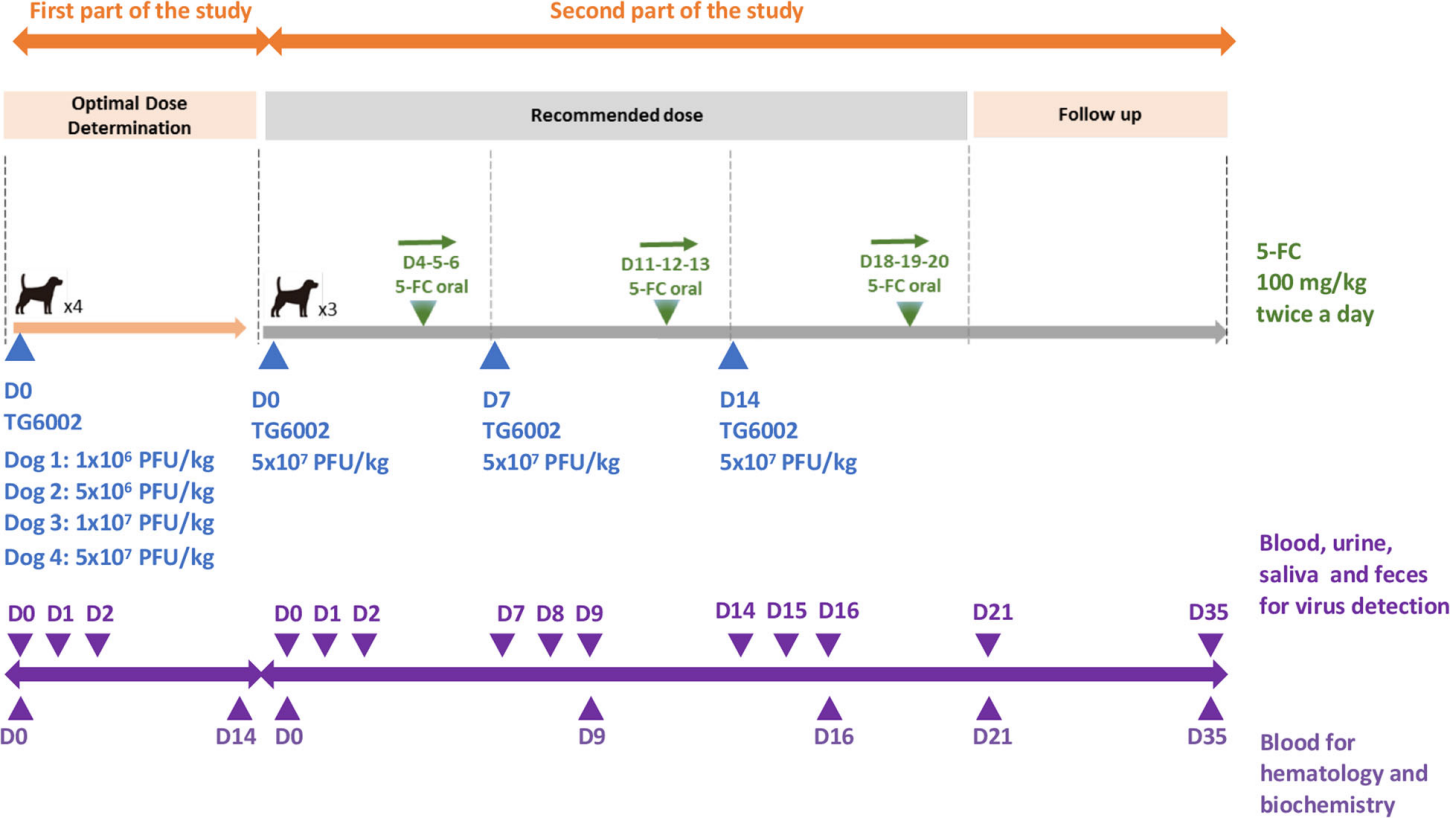
Study chart and sample collection for safety evaluation of TG6002 in dogs. The first part of the study was conducted using four dogs to determine the maximal tolerated dose of TG6002. The second part of the study included three new dogs that were injected with TG6002 at the maximal dose determined in the first part of the study. The dogs were given three intramuscular injections of TG6002 with one-week intervals between injections. Four days after each injection, 5-FC was orally administered for three days. Samples collections for virus detection, complete blood count and blood chemistry are indicated

The first part of the study consisted of determining the MTD in four dogs, with each dog receiving a single different dose of TG6002 by intramuscular injection. The second part of the study assessed tolerability of several injections of TG6002 at the MTD, identified in the first part of the study, in combination with 5-FC administration. Intramuscular injections were chosen to mimic intratumoral route of administration in healthy dogs. The overall schedule of the study is shown in Fig. 5.

In the first part of the study, four dogs were randomized for single intramuscular injection of TG6002. The doses chosen in this study were similar to the doses administered in a clinical trial using TG6002 in human patients with advanced gastrointestinal tumors (NCT03724071) [58]. Dog 1 received 1 × 10^6^ PFU/kg, Dog 2 received 5 × 10^6^ PFU/kg, Dog 3 received 1 × 10^7^ PFU/kg and Dog 4 received 5 × 10^7^ PFU/kg, diluted with NaCl 0.9%. A single intramuscular (quadriceps or lumbar muscle) injection with a maximal volume of 0.5 ml/kg per site was performed in each dog. Multiple site injections were performed if the volume was over 0.5 ml/kg. Injections were performed in a dedicated room of the laboratory animal facility and outside the room housing the dogs. To detect any side effects, injections were performed early in the morning to allow for observations and administrations between dogs were spaced by 7 days.

Dogs were evaluated once-a-day by a physical examination over 14 days, after the injection of TG6002. Complete blood counts were performed using a Procyte Hematology analyzer (IDEXX Laboratory Inc., Westbrook, Maine, United States) before and 14 days after the injection of TG6002. Biochemistry analyses were performed before and 14 days after the injection of TG6002 using a Catalyst Biochemistry analyzer (IDEXX Laboratory Inc., Westbrook, Maine, United States).

Body fluid samples (blood, saliva, urine and feces) were collected at days 0 (before virus injection), 1 and 2 and analysed to assess viral shedding. The MTD was defined as the highest dose of TG6002 that did not cause major side effects.

Three new dogs (Dog 5, Dog 6, Dog 7) were randomized for evaluating the tolerability to multiple injections combined with 5-FC. Dogs received three intramuscular injections of TG6002 at day 0, 7 and 14 at the defined MTD. Four days after each injection, 5-FC (Toronto Research Chemicals, North York, ON, Canada) was orally administered at a dose of 100 mg/kg twice a day for 3 days. Injections were performed in a dedicated room of the laboratory animal facility and outside the room housing the dogs. To detect any side effects, injections were performed early in the morning to allow for observations and administrations between dogs were spaced by 7 days.

Dogs were evaluated once-a-day by physical examination until 2 weeks after the last administration of 5-FC. Complete blood counts were performed at day 0, 9, 16, 21 and 35 using a Procyte Hematology analyzer (IDEXX Laboratory Inc., Westbrook, Maine, United States). Biochemistry analyses were performed at days 0, 16 and 35 using a Catalyst Biochemistry analyzer (IDEXX Laboratory Inc., Westbrook, Maine, United States).

Bodily fluid samples (blood, saliva, urine and feces) were collected at day 0 (before virus injection), 1, 2, 7 (before second virus injection), 8, 9, 14 (before third virus injection), 15, 16, 21, and 35 and analysed to assess viral shedding.

### Adverse events

Assessment of adverse events was performed according to the Veterinary Cooperative Oncology Group Common Terminology Criteria for Adverse Events v1.1 guidelines [57]. Adverse events were monitored throughout the study by daily physical examination, complete blood count and biochemistry analyses.

### Viral shedding

Viral shedding was evaluated for both part of the study, on blood, saliva, urine and feces collected before and during the 48 h after each injection of TG6002. Additional evaluations were performed at day 21 and day 35 during the second part of the study (Fig. 5). Five milliliters of blood were collected in an EDTA tube, saliva samples were taken with buccal swabbing (Universal viral transport kit, Becton Dickinson, Franklin Lakes, New Jersey, United States), 5 ml of urine were collected by ultrasonography guided vesical puncture and placed in a sterile Falcon tube, and one gram of feces was transferred to a sterile Falcon tube. All samples were stored at − 80 °C until analysis. Viral shedding was evaluated by viral titration by plaque assay on CEF (for blood, saliva and urine) and by qPCR assay (for blood, saliva, urine and feces).

### Viral titration by plaque assay on CEF

Viral titration by plaque assay was performed on CEF, each sample (blood, saliva, urine) was tested after dilution of 100 μl of sample in phosphate buffered saline supplemented with 1% fetal calf serum (FCS, Life Technologies, Carlsbad, California, United States) and 1% cations (magnesium acetate 100 μg/mL, calcium chloride 100 μg/mL, Merck, Darmstadt, Germany) up to 1 ml. Diluted samples were sonicated three times for 5 min at room temperature and were titrated in triplicate on CEF by plaque assay as previously described [11].

### Viral quantification by qPCR assay

DNA was extracted from 50 μl of the whole blood, saliva, and urine samples using an automatic MagMax96 Deep Well (Life Technologies, Carlsbad, California, United States). Eight hundred milligrams of feces sample were diluted in 20 ml of phosphate buffered saline, sonicated and centrifuged for 4 min at 1400 rpm. DNA extraction was performed on 50 μl of sample using the MagMax 96 Viral RNA Isolation kit (Life Technologies, Carlsbad, California, United States) and the automatic MagMax96 Deep Well (Life Technologies, Carlsbad, California, United States). qPCR amplification was performed with the Multiplex Quantitect kit (Qiagen, Hilden, Germany) and was based on primers designed on VACV ITR region sequence (forward primer: CGATGATGGAGTAATAAGTGGTAGGA, reverse primer: CACCGACCGATGATAAGATTTG, probe: ACTGATTCCACCTCGGG). A standard curve was generated for absolute quantification by using the plasmid pTG15212 which contains the cloned VACV ITR sequence at both ends of the virus and located between position 6352 to 8180 (relative to Gene Bank sequence U94848) in the 5’region and position 169,909 to 171,737 in the 3′ region of the viral genome. The ratio of plasmid/virus was two copies of pTG15212 for one copy of TG6002. Absolute quantification was performed by using a standard curve of pTG15212 plasmid. Positive controls were included with spiked samples of 10 μl of the plasmid pTG15212 (10,000 copies) solution. All samples were run in triplicate. The limit of detection for analysis was 150 copies/ml for whole blood, 300 copies/ml for urine and 36 copies/mg for feces. It was not possible to define detection limits for saliva, since the amount of collected saliva was exceedingly low.

### Histological and immunohistochemical analyses

Histological analyses were performed on Hematoxylin-Eosin-Saffron stained tissue sections. Immunohistochemical analyses were performed on kidney and liver samples using a rabbit anti-VACV polyclonal antibody (dilution 1/500) (B65101R, Meridian Life Science, Memphis, Tennessee). Immunohistochemical analyses were performed using a DXT automat (Ventana Medical Systems, Roche Diagnostics, Basel, Switzerland) with the streptavidin-biotin-peroxidase complex method with 3,3′-Diaminobenzidine as a substrate and hematoxylin counterstaining Formalin-fixed. As previously described, a canine mammary adenocarcinoma explant infected with TG6002 was used as positive control [13].

## Supplementary information

**Additional file 1.** Biochemical analyses of dogs after single intramuscular injection of TG6002. No significant anomalies were noted. Bold numbers refer to values outside the reference interval.

**Additional file 2.** Biochemical analyses of dogs after three intramuscular injections of TG6002. No significant anomalies were noted. Bold numbers refer to values outside the reference interval.

**Additional file 3.** Detection of virus shedding by plaque assay after single intramuscular injection of TG6002. Infectious VACV was not detected in blood, urine and saliva samples by plaque assay method from dogs treated with escalating doses of TG6002. All samples were run in triplicate.

**Additional file 4.** Detection of virus shedding by plaque assay after three intramuscular injections of TG6002. Infectious VACV was not detected in blood, urine and saliva samples by plaque assay from dogs treated with three successive injections of TG6002 at 5 × 10^7^ PFU/kg. All samples were

**Additional file 5.** Detection of virus shedding by qPCR assay after a single intramuscular injection of TG6002. VACV DNA was not detected in blood, urine and saliva samples by qPCR assay from dogs treated with escalating doses of TG6002. All samples were run in triplicate.

**Additional file 6.** Detection of virus shedding by qPCR assay after three intramuscular injections of TG6002. VACV DNA was not detected in blood, urine and saliva samples by qPCR assay from dogs treated with three successive injections of TG6002 at 5 × 10^7^ PFU/kg. All samples were run in triplicate.

## Data Availability

The datasets used and/or analysed during the current study are available from the corresponding author on reasonable request.

## Abbreviations

CEF: Chicken embryo fibroblast
FCS: Fetal calf serum
MTD: Maximal tolerated dose
TK: Thymidine kinase
RR: Ribonucleotide reductase
VACV: Vaccinia virus
5-FC: 5-Fluorocytosine
5-FU: 5-Fluorouracil
